# LMWP (S3-3) from the Larvae of *Musca domestica* Alleviate D-IBS by Adjusting the Gut Microbiota

**DOI:** 10.3390/molecules27144517

**Published:** 2022-07-15

**Authors:** Siyuan Peng, Xiwen Ling, Wenjing Rui, Xiaobao Jin, Fujiang Chu

**Affiliations:** 1 Department of Clinical Laboratory, Third Affiliated Hospital of Guangzhou Medical University, No. 63 Duobao Road, Liwan District, Guangzhou 510150, China; 2022683061@gzhmu.edu.cn; 2 Guangdong Provincial Key Laboratory of Pharmaceutical Bioactive Substances, Guangdong Pharmaceutical University, Guangzhou 510006, China; 18122415951@163.com (X.L.); ruiwenjin2019@163.com (W.R.); jin1960gdpu@163.com (X.J.)

**Keywords:** larvae of *Musca domestica*, D-IBS, GI dysfunction, 5-HT, gut microbiota

## Abstract

Diarrhea-based Irritable Bowel Syndrome (D-IBS) and diarrhea are both associated with ecological imbalance of the gut microbiota. Low Molecular Weight Peptides (LMWP) from the larvae of *Musca domestica* have been shown to be effective in the treatment of diarrhea and regulation of gut microbiota. Meanwhile, the single polypeptide S3-3 was successfully isolated and identified from LMWP in our previous studies. It remains unclear exactly whether and how LMWP (S3-3) alleviate D-IBS through regulating gut microbiota. We evaluated the gut microbiota and pharmacology to determine the regulation of gut microbiota structure and the alleviating effect on D-IBS through LMWP (S3-3). The rates of loose stools, abdominal withdrawal reflex (AWR) and intestinal tract motility results revealed that LMWP (S3-3) from the larvae of *Musca domestica* had a regulating effect against diarrhea, visceral hypersensitivity and gastrointestinal (GI) dysfunction in D-IBS model mice. Additionally, 16S rRNA gene sequencing was utilized to examine the gut microbiota, which suggests that LMWP induce structural changes in the gut microbiota and alter the levels of the following gut microbiota: *Bacteroidetes*, *Proteobacteria* and *Verrucomicrobia*. LMWP putatively functioned through regulating 5-HT, SERT, 5-HT2AR, 5-HT3AR and 5-HT4R according to the results of ELISA, qRT-PCR and IHC. The findings of this study will contribute to further understanding how LMWP (S3-3) attenuate the effects of D-IBS on diarrhea, visceral hypersensitivity and GI dysfunction.

## 1. Introduction

Irritable Bowel Syndrome (IBS) is a functional disorder of the GI tract that is characterized by stomach ache, bloating and altered bowel behavior. Notably, the global prevalence of IBS was estimated to be around 7–30% [1]. The latest epidemiological study shows that the global prevalence of IBS is 11.2%, which also showed that D-IBS, C-IBS, M-IBS and U-IBS subtypes accounted for 23.4%, 22.0%, 24.0% and 22.2% of patients with IBS, respectively [2]. Although IBS is not a life-threatening disease, it seriously affects the normal life of patients and also creates economic burden. Among the four types of IBS, D-IBS is the most common. Young and middle-aged groups (18–59 years old) are the main patients who have the disease [3]. Stress in life comes from various sources, which act as predisposing risk factors for the development of irritable bowel syndrome (IBS). Physical stressors can affect visceral events. Additionally, IBS patients are at a greater risk of comorbidities, incur higher overall medical costs and have a reduced quality of life. Existing evidence suggests that the pathogenesis of IBS is multifaceted, including immunological, genetic and environmental influences [1]. Nonetheless, the etiology of IBS remains unclear, although accumulating evidence suggests that visceral hypersensitivity, impaired gastrointestinal motility, disturbance of microbial equilibrium, inflammation and/or intestinal infection may all be biological abnormalities associated with the condition [4,5,6]. Additionally, IBS can be classified into four groups based on clinical symptoms: Diarrhea-based Irritable Bowel Syndrome (D-IBS), Constipation-based Irritable Bowel Syndrome (C-IBS), Mixed Irritable Bowel Syndrome (M-IBS) and Undefined Irritable Bowel Syndrome (U-IBS) [7], with D-IBS being the most common. Notably, antispasmodic drugs, anticholinergics, antidiarrheal agents, visceral analgesics and antipsychotics are currently used to treat D-IBS [8]. Furthermore, treatment of D-IBS is primarily symptomatic relief medication, although it is associated with adverse effects that may have severe psychiatric consequences for patients [9]. Having similar efficacy, natural drugs have safer and less adverse effects as compared to synthetic chemical drugs. Therefore, it is critical to identify new therapeutic approaches capable of changing the composition of gut microbiota, enhancing the metabolism of neuroendocrine transmitters, decreasing visceral vulnerability and having a comprehensive regulatory effect on gut microbiota.

Currently, *Musca domestica* (housefly, Diptera: Muscidae) larvae are regarded as excellent sources of high-quality protein, polyunsaturated fats, saccharides, vitamins, minerals and other nutrients, for both human consumption and animal feed. In China, Li Shizhen demonstrated the role of these larvae in alleviating malnutrition in infants (stool induration or diarrhea) [10].

These peptides from natural sources were identified and had an alleviative effect on disease. A recent study showed that a spider-venom peptide with multi-target activity on sodium and calcium channels alleviates chronic visceral pain in IBS [11]. Bioactive fish collagen peptides weaken intestinal inflammation by orienting colonic macrophages phenotype through mannose receptor activation [12]. *Musca domestica* cecropin, a novel antimicrobial peptide, possessed potential antibacterial, anti-inflammatory, immunological functions and had a protective effect on colonic mucosal barrier injury caused by *Salmonella typhimurium*, which were reported by our laboratory [13].

Moreover, our previous studies reported that LMWP from the larvae of *Musca domestica* had antidiarrheal effects via regulation of the gut microecology and LMWP (S3-3) that were successfully isolated and identified [14]. Notably, the gut microbiota consists of more than 100 trillion microbes residing within the GI tract. Furthermore, extensive research has demonstrated that gut microbiota play a vital role in maintaining human health [15,16,17]. Additionally, disturbance of microbial equilibrium or dysbiosis has been shown to be closely related to multiple disorders including D-IBS, obesity, hyperlipidemia, atherosclerosis and numerous types of cancer. Therefore, the present study hypothesized that LMWP (S3-3) from the larvae of *Musca domestica* would be effective in alleviating diarrhea and D-IBS.

Therefore, this study aimed to determine the in vivo effect of LMWP (S3-3) from larvae of *Musca domestica* in alleviating D-IBS and gut microbiota imbalance. The psychosocial stress (restraint) model better simulates the pathogenesis of human IBS and gastrointestinal dysfunction [18,19]. Similar studies also verified the high efficacy and sustainability of the model, allowing for a better understanding of the pathological process as well as the vulnerability and triggering factors in D-IBS [20,21,22].

## 2. Materials and Methods

### 2.1. Preparation of LMWP (S3-3) from the Larvae of Musca domestica

LMWP (S3-3) from the larvae of *Musca domestica* (purity 94.70%, molecular weight: 1069.4391 Da; the 10 amino acid sequences of S3-3 were Val-Tyr-Arg-Asp-Asn-Val-Leu-Phe-Gln-Ala) were prepared as described in our previous study [14].

The laboratory strain of *Musca domestica* was obtained as a kind gift from the Guangdong Provincial Center for Disease Control and Prevention CDC, China. The larvae of *Musca domestica* were then dried using conventional drying systems. Briefly, third-instar larvae of *Musca domestica* were collected, washed for 3 h with running tap water, frozen for 2 h at −20 °C and sun dried for 6 h.

Following that, 1000 g of dried larvae were fried until they turned light yellow and then sifted using 40-mesh sieves. The larvae was fried to modulate the therapeutic properties of treated herbal medicines, i.e., enhancing efficacy, reducing toxicity or side effects, which is a kind of processing of Chinese medicine [23,24,25]. Following that, the dried powder of larvae of *Musca domestica* (10.0 g) was immersed in deionized water (250 mL) in a beaker for 30 min [14]. The samples were then simmered for 10 min before removing the supernatant and centrifuging at 12,000 r/min for 10 min. In addition, an equal volume of deionized water was added, and the same procedure was repeated. Following that, both supernatants with a molecular weight of <30 kD were collected by ultrafiltration technology (ultrafiltration membrane with a molecular weight cut-off of 30 kD) and lyophilized [15]. Finally, the supernatants were freeze-dried and LMWP were collected from larvae of *Musca domestica*.

A suitable amount (800 mg) of the LMWP powder was accurately weighed and dissolved in 2.0 mL of ultra-pure water, and the mixture was filtered on a 0.22 μm filter. Then, the LMWP powder was fractionated according to their molecular masses by using gel-filtration chromatography (GFC) on a column packed with SuperdexTM 30 and eluted with deionized water at a flow rate of 0.6 mL/min. Each eluate (3 mL) was collected and monitored at 280 nm, and the fractions (1 mL) were collected at a flow of 0.25 mL/min. Based on gel-filtration chromatography, the components of LMWP (S3-3) (t = 3.0 min) were further separated by RP-HPLC. YMC-Pack C4-HG columns (4.6 mm × 250 mm, 10 μm) were separately used for S3-3 separation. The fractions were automatically collected at a flow rate of 1 mL/min and dried by centrifugation under vacuum. The identification of the LMWP (S3-3) from the larvae of *Musca domestica* was performed in our previous study [9]. The purity of the fractions was determined by HPLC, the molecular weight was identified by MALDI-TOF spectrometer and the N-terminal sequences were determined using Edman degradation (Supplementary Figures S1–S3, Supplementary Table S1).

### 2.2. Animals and Experimental Design

**Figure 1:**
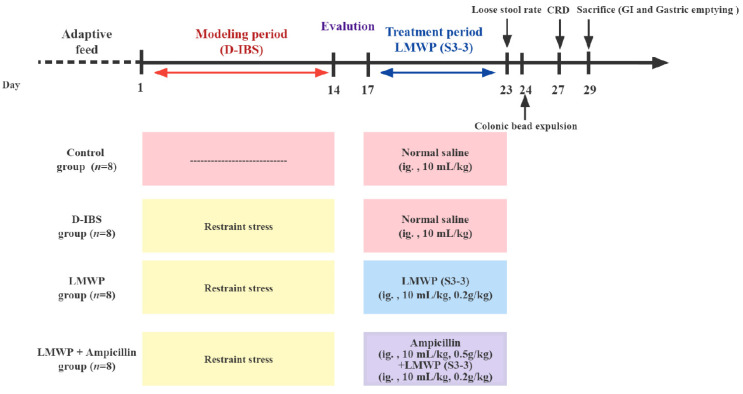
Timeline of experimental procedures.

A total of 32 male SPF C57BL/6J mice were provided by Medical Laboratory Animal Center, Guangdong Province (Guangzhou, China approval number SCXK (Yue) 2013-0002). The 6-week-old male mice were then housed in a specific-pathogen-free facility (room temperature 22 ± 2 °C and 12/12 h light/dark cycle) for 7 days (eating and drinking ad libitum). This was conducted in accordance with the guidelines by Care and Use of Experimental Animals. Additionally, the use of animals was approved by the Guangdong Pharmaceutical University and the Guangdong Pharmaceutical University Animal Care and Use Committee, China. Animal grouping design is shown in Figure 1. The 32 C57/BL6J mice (20 ± 2 g) were randomly assigned to four groups (*n* = 8) as shown in Figure 1: the Control, D-IBS, LMWP and LMWP + ampicillin groups. Among these groups, the LMWP + ampicillin group was established to identify the role of gut microbiota in D-IBS. After confirming the successful establishment of the D-IBS model, mice in the Control and D-IBS groups were given 10 mL/kg of Control saline, the LMWP group was intragastrically treated with 0.2 g/kg of LMWP (S3-3) from the larvae of *Musca domestica* (10 mL/kg) and those in the LMWP + ampicillin group received 500 mg/kg of ampicillin (10 mL/kg) and 0.2 g/kg of the LMWP (S3-3) from the larvae of *Musca domestica* (10 mL/kg), through the intragastric route, and all the mice were treated for 7 days. Thereafter, at the end of the therapy cycle, five fecal samples were randomly collected from each group for 16S rDNA gene sequencing.

### 2.3. Introduction of D-IBS in Mice

The D-IBS model was then established by chronic restraint stress (24 mice were immobilized using a plastic restrainer for a duration of 1 h daily) for 14 days [18,19], in D-IBS, LMWP and LMWP + ampicillin groups. An abdominal withdrawal reflex (AWR) score ≥ 2 points and a loose stool rate ≥ 0.5 revealed that the D-IBS mice model was successfully established. Physical stressors can affect visceral events. Animals subjected to stress result in abnormal intestinal motility and visceral hypersensitivity. Stress-induced IBS models, e.g., restraint stress, could largely mimic IBS symptoms from intestinal motility to visceral sensitivity [20,21,22].

### 2.4. Determination of the Rate of Loose Stool

The total number of stools and loose stools was determined using the filter paper imprinting method [26,27]. The mice were placed in individual cages and the cage floor was covered with filter paper. The number and morphology of the stools were recorded for 6 h. The loose stools were classified into five grades based on the diameters of stain formed by loose stools on the filter paper: Grade 1 (0 < 1 cm), Grade 2 (1~2 cm), Grade 3 (2~3 cm), Grade 4 (3~4 cm) and Grade 5 (4~5 cm). Therefore, the rate of loose stool (%) = number of loose stools/total number of stools × 100%. Loose stool grade was defined as the calculated mean of the diameters of stain formed by loose stools on the filter paper. Loose stool index was determined as follows: loose stool index = rate of loose stool × loose stool grade.

### 2.5. The Abdominal Retraction Reflex (AWR) Score

The mice were subjected to a 24 h fast before inserting the 6F catheter, after paraffin oil lubrication, through the anus. A double-lumen balloon was then placed about 2.0 cm from the anus. Then, mice were placed inside a restraint device. After adapting to the new environment, the mice were gradually injected with water to dilate the balloon. The dilation capacity was 0.25 mL, 0.35 mL and 0.50 mL and each rectal dilation lasted for 30 s. The procedure was repeated thrice and the mean value was calculated. Finally, the AWR scores were determined using the following scale: 0, no behavioral response to Colorectal Distension (CRD); 1, brief head movement followed by immobility; 2, contraction of abdominal muscles; 3, lifting of the abdomen; 4, body arching and lifting of pelvic structures. These methodologies were performed and modified according to those previously described [28].

### 2.6. Colonic Bead Expulsion Test

Under anesthesia, glass beads (2 mm in diameter) were inserted into the rectum (about 3 cm from the anus). The mice were then placed in a cage (1 mouse/cage) with no access to food or water. After the mice were fully awake (the standard was that the mice could turn over freely and climb up), the study began by observing the time to bead ejection. These methodologies were performed and modified as previously described [29,30].

### 2.7. Upper GI Transit

The mice in each group were fasted for 24 h and then orally administered with 0.2 mL of a suspension of the charcoal meal (10% charcoal in 5% gum arabic). The mice were sacrificed 20 min after receiving the charcoal meal. The small intestine was removed en bloc and the length of the small bowel and the distance traveled by the charcoal meal were then measured for each mouse. The ratio of the distance traveled by the charcoal meal to the total length of the small bowel was then used as the upper GI transit. These methodologies were performed and modified according to those previously described [31,32].

### 2.8. Gastric Emptying

The mice in each group received 0.2 mL of a suspension of the charcoal meal (10% charcoal in 5% gum arabic) and were sacrificed after 20 min. This was followed by abdominal dissection and ligation of the gastric cardia and pylorus. The stomach was then dried using a filter paper and the full weight was obtained. Following that, the stomach was cut along its bend before washing off the stomach contents and drying with filter paper. Gastric emptying (%) was calculated using the following formula: Gastric emptying (%) = (full weight of stomach − dry weight of stomach/weight of suspension of charcoal meal) × 100%. These methodologies were performed and modified according to those previously described [32,33].

### 2.9. Histological Analysis

The colonic tissues from 8 mice were examined in each group. Colon sections were excised to assess histological changes in the colon and were gently irrigated with normal saline to dislodge the intestinal contents. They were then fixed immediately at 4 °C overnight in 4% paraformaldehyde solution. Following three washes in tap water, they were dehydrated with serial ethanol concentrations. They were rinsed with xylene, paraffin-embedded, sliced into 4 μm sections and stained with Hematoxylin and Eosin (H&E). They were then examined under a light microscope and photomicrographs of the sections were taken using a digital camera (DFC495 Digital camera Leica, Leica Microsystems, Wetzlar, Germany).

### 2.10. Enzyme-Linked Immunoassay (ELISA)

Blood samples were centrifuged at 3000 rpm for 10 min at 4 °C. Following that, serum was collected and immediately frozen in liquid nitrogen before being stored at −80 °C for further analysis. Additionally, the distal colon was homogenized in cold PBS. Following this, the frozen colonic tissues were homogenized and lysed in the tissue lysis buffer, followed by centrifugation at 12,000 rpm for 10 min at 4 °C. The supernatant was then collected. 5-HT, a critical signaling molecule in the gut, activated both intrinsic and extrinsic primary afferent neurons to initiate peristaltic and secretory reflexes. The levels of 5-HT in serum and colonic tissues were determined using an ELISA kit (Shanghai MLBIO Biotechnology Co., Ltd., Shanghai, China), and the operation steps of the kit were performed following the manufacturer’s instructions.

### 2.11. Real-Time Quantitative PCR Detection

RNA from the distal colon was extracted. Total RNA isolation and cDNA synthesis were accomplished using the Trizol reagent (Accurate Biology Co., Ltd., Changsha, China) and the PrimeScript^TM^ RT reagent Kit with a gDNA Eraser (Accurate Biology Co., Ltd., Changsha, China), respectively. The mRNA levels of particular genes were then determined by real-time PCR using SYBR Green Pro Taq HS Premix (Accurate Biology Co., Ltd., Changsha, China) in the CFX Connect fluorescence quantitative PCR detection system (BIO-RAD, Hercules, CA, USA). Notably, the 20 µL PCR reaction mixture comprised 10 μL 2× SYBR Green Pro Taq HS Premix, 0.4 μL Forward Primer (10 μM), 0.4 μL Reverse Primer (10 μM), 2 μL reaction solution (cDNA) and 7.2 μL RNase-free water. Additionally, the following protocol was used for the Shuttle PCR: Stage 1 was the initial denaturation of one cycle at 95 °C for 30 s; Stage 2 was the PCR reaction of 40 cycles at 95 °C for 5 s, and 60 °C for 30 s; Stage 3 was the dissociation step. The data were analyzed using the comparative threshold cycle (Cq) method and normalized to an endogenous reference, Glyceraldehyde-3-phosphate Dehydrogenase (GAPDH). 5-HT2AR, 5-HT3AR and 5-HT4R were 5-HT receptors, and SERT was a 5-HT reuptake transporter, which was involved in the reuptake and inactivity of 5-HT. The relative expression levels of genes associated with gastrointestinal movement (5-H2AR, 5-HT3AR, 5-HT4R, SERT) were then determined in colon tissues and calculated using the 2^−∆∆CT^ method. The primers used in this experiment are listed in Table 1.

### 2.12. Immunohistochemistry (IHC)

Mice were sacrificed at the end of the experiments. Colon tissues were then isolated, embedded on paraffinized blocks and cut into 4 µm-thick sections, individually, using a microtome (Leica, Wetlar, Germany). Next, the sections were incubated with anti-5-HT1AR rabbit polyclonal antibody, anti-5-HT2AR rabbit polyclonal antibody, anti-SERT polyclonal antibody (1:50) (Sangon Biotech, Shanghai, China) and anti-5-HT4AR rabbit polyclonal antibody (1:100) (Bioss, Beijing, China) overnight at 4 °C in a dilution ratio of 1:100 using the BondTM Primary Antibody Diluent (Servicebio, Wuhan, China). On the next day, the sections were incubated for 1 h with horseradish peroxidase 4-layered goat anti-rabbit secondary antibodies at 37 °C (Sangon Biotech) according to the manufacturer’s instructions. Finally, the sections were treated with diaminobenzidine (DAB) solution (Servicebio, Wuhan, China) and visualized under a microscope (NIKON, Eclipse, Ci, Tokyo, Japan). We measured the integrated optical density (IOD) from at least three fields of each slice using the Image pro-plus 6.0 software (Media Cybernetics, Bethesda, MD, USA), which could accurately reflect the complete expression of the proteins in immunohistochemical staining.

### 2.13. Gut Microbiota Analysis

The fresh stool was collected from the colons of mice after being sacrificed and immediately frozen at −80 °C. Additionally, bacterial genomic DNA was extracted from frozen stool samples using the Qiagen QIAamp DNA stool Mini Kit (Hilden, Germany) according to the manufacturer’s instructions. Following that, the 16S rRNA in the V3-V4 region (341F-805R, F: GATCCTACGGGAGGCAGCA; R: GCTTACCGCGGCTGCTGGC) was amplified via thermal cycling consisting of initial denaturation step at 98 °C for 1 min, followed by 30 cycles of denaturation at 98 °C for 10 s, annealing at 50 °C for 30 s and elongation at 72 °C for 60 s and a final hold at 72 °C for 5 min. Purification was subsequently performed using the MinElute Gel Extraction Kit (Qiagen, Shanghai, China) and samples with 400–450 bps were chosen for further experiments. Sequencing libraries were generated using the NEB Next Ultra DNA Library Prep Kit for Illumina (NEB, Ipswich, MA, USA), following the manufacturer’s instructions, and index codes were added. The PCR results were then subjected to high-throughput sequencing on an Illumina HiSeq2500 platform (Biomarker Technologies Co., Ltd., Beijing, China). Additionally, in the Greengenes database (13.5 version) (Lawrence Berkeley National Laboratory, Berkeley, CA, USA), USEARCH software (10.0 version) (Robert Edgar, Tiburon, CA, USA) was used to select OTUs and the RDP classifier (2.2 version) was used to annotate taxonomic information for each representative sequence. The alpha diversity indices among groups were compared after the sequences were rarefied to control for depth. The QIIME software package was also used to perform UniFrac distance-based Principal Component Analysis (PCA). Permutational Multivariate Analysis of Variance (PERMANOVA) was also performed. The Z score, the value corresponding to the heatmap, was obtained after the relative abundance of each row of species had been standardized in the heatmap of the gut microbiota at the genus level. Finally, Linear discriminant analysis Effect Size (LEfSe) analysis was performed using Metastats software to identify the biomarker species, Linear Discriminant Analysis (LDA) highlighting significant biomarker species among each group’s microbiota, LDA threshold of >4. The relative abundance of significant biomarker species, obtained in gut microbiota from the LEfSe results, was compared in each group.

### 2.14. Statistical Analysis

SPSS Statistics 17.0 software (IBM, Armonk, NY, USA) was used to perform statistical analyses. All data were presented as mean ± SD and multiple comparisons were performed using one-way Analysis of Variance (ANOVA). *p* values less than 0.05 were considered statistically significant. Spearman’s correlation was measured to demonstrate the relationships between parameters, the correlation coefficient was always in the range of +1 to −1. The correlations between gut microbial biomarkers and the phenotype were corrected by False Discovery Rate (FDR), which was calculated through the Benjamini–Hochberg (BH) method.

## 3. Results

### 3.1. Effects of LMWP (S3-3) from Larvae of Musca domestica on Physiological Conditions and the Frequency of Loose Stools in D-IBS Mice

**Figure 2:**
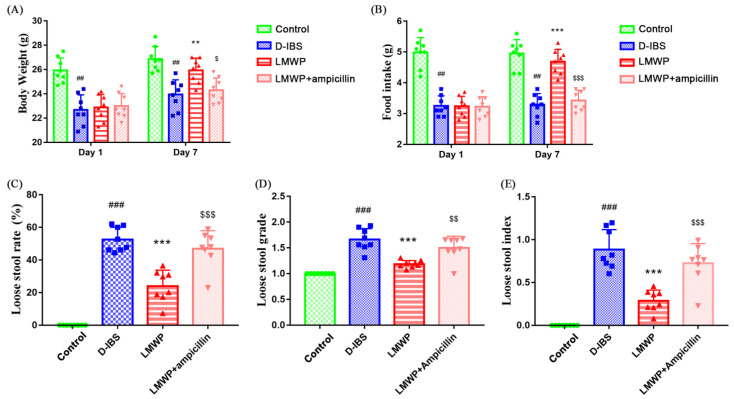
LMWP (S3-3) from the larvae of *Musca domestica* affects body weight, food intake and diarrhea in D-IBS mice. (**A**) Body weight on day 1 and day 7 in the treatment period. (**B**) Food intake on day 1 and day 7 in the treatment period. (**C**–**E**) Loose stool rate, loose stool grade and loose stool index. Values are presented as means ± SD (*n* = 8). ^##^ *p* < 0.01 and ^###^ *p* < 0.001 compared with Control, ** *p* < 0.01 and *** *p* < 0.001 compared with D-IBS, ^$^ *p* < 0.05, ^$$^ *p* < 0.01 and ^$$$^ *p* < 0.001 compared with LMWP (S3-3) from the larvae of *Musca domestica*.

After 14 days of D-IBS induction, D-IBS mice had significantly lower body weight and food intakes than the Control group. On day 1 of treatment, there were no statistically significant differences in initial body weight and food intake between the D-IBS, LMWP and LMWP + ampicillin groups. After 7 days of administration of LMWP (S3-3) from larvae of *Musca domestica*, the body weight and food intake in the LMWP group were significantly greater than in the D-IBS group. After seven days of treatment, the body weight and food intake in the LMWP + ampicillin group were significantly lower than in the LMWP group (Figure 2A,B). The D-IBS group had a higher loose stool rate, loose stool grade and loose stool index than the Control group. Additionally, when compared to the D-IBS group, the LMWP group demonstrated significant improvement in loose stool rate, loose stool grade and loose stool index. Additionally, as demonstrated in Figure 2C–E, the LMWP + ampicillin group had increased loose stools rate, loose stool grade and loose stool index compared to the LMWP group.

### 3.2. Effects of LMWP (S3-3) from Larvae of Musca domestica on Gastrointestinal Motility and Visceral Sensitivity in Mice

**Figure 3:**
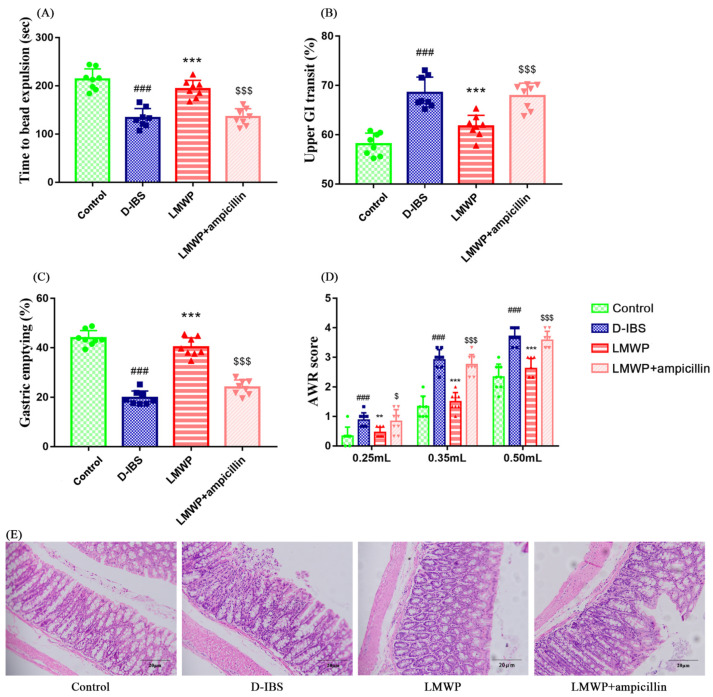
LMWP (S3-3) from the larvae of *Musca domestica* affect intestinal tract motility, visceral hypersensitivity and colonic histology in D-IBS mice. (**A**) Time to bead expulsion. (**B**) Upper gut transit. (**C**) Gastric emptying. (**D**) AWR scores. (**E**) Morphology found in the colon. Values are presented as means ± SD (*n* = 8). ^###^ *p* < 0.001 compared with Control, ** *p* < 0.01 and *** *p* < 0.001 compared with D-IBS, ^$^ *p* < 0.05 and ^$$$^ *p* < 0.001 compared with LMWP (S3-3) from the larvae of *Musca domestica*.

Intestinal transit in mice was evaluated using the time to bead expulsion, upper GI transit and degree of gastric emptying. There was an increase in upper GI transit and a significant decrease in the efflux time of glass beads as well as in the degree of gastric emptying in mice in the D-IBS group compared to those in the Control group during the modeling process (Figure 3A–C). This indicated that the frequency of GI transport increased significantly after the modeling process. Additionally, mice in the LMWP group exhibited a slower upper GI transit and an increased efflux time for the glass beads, as well as a greater degree of gastric emptying, compared to those in the D-IBS group. However, there were significant differences in upper GI transit, efflux time of glass beads and degree of gastric emptying between the LMWP + ampicillin and LMWP groups. LMWP + ampicillin group had increased upper GI transit and decreased efflux time of glass beads, as well as a greater degree of gastric emptying than the LMWP group.

Additionally, visceral sensitivity was assessed using the abdominal uplift and back arch volume thresholds. Compared to the Control group, there was a significant increase in the AWR scores of the D-IBS group, demonstrating increased visceral sensitivity following the modeling process. However, as compared to the D-IBS group, the AWR scores decreased significantly following treatment with LMWP (S3-3) from larvae of *Musca domestica* (Figure 3D), suggesting meliorative visceral sensitivity following treatment with LMWP (S3-3) from larvae of *Musca domestica*. In comparison to the LMWP group, the LMWP + ampicillin group showed significantly higher AWR scores.

### 3.3. Effect of LMWP (S3-3) from Larvae of Musca domestica on Colonic Histological Assessment

Figure 3E showed that the colon tissues of the Control, D-IBS, LMWP and LMWP + ampicillin groups were normal. The mucosa was normal and complete, and neatly arranged villi were observed; the muscle layer was even and moderate, and colonic epithelial cells were arranged regularly in each group. Moreover, no significant pathological changes were observed in any group.

### 3.4. Effect of LMWP (S3-3) from Larvae of Musca domestica on the Expression of Genes and Proteins Involved in 5-HT-Related Pathways

**Figure 4:**
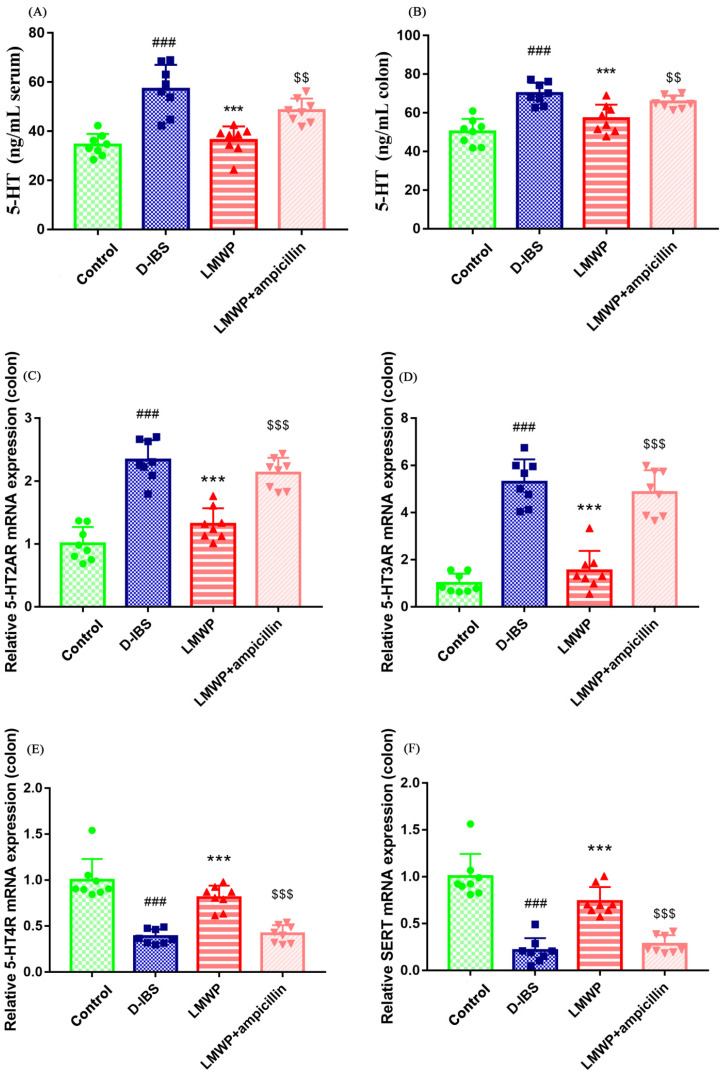
LMWP (S3-3) from the larvae of *Musca domestica* affect 5-HT, 5-HT2AR, 5-HT3AR, 5-HT4R and SERT in D-IBS mice. (**A**,**B**) 5-HT levels in serum and colon. (**C**–**F**) The relative expression of 5-HT2AR, 5-HT3AR, 5-HT4R and SERT mRNA in the colon. Values are presented as means ± SD (*n* = 8). ^###^ *p* < 0.001 compared with Control, *** *p* < 0.001 compared with D-IBS, ^$$^ *p* < 0.01 and ^$$$^ *p* < 0.001 compared with LMWP (S3-3) from the larvae of *Musca domestica*.

**Figure 5:**
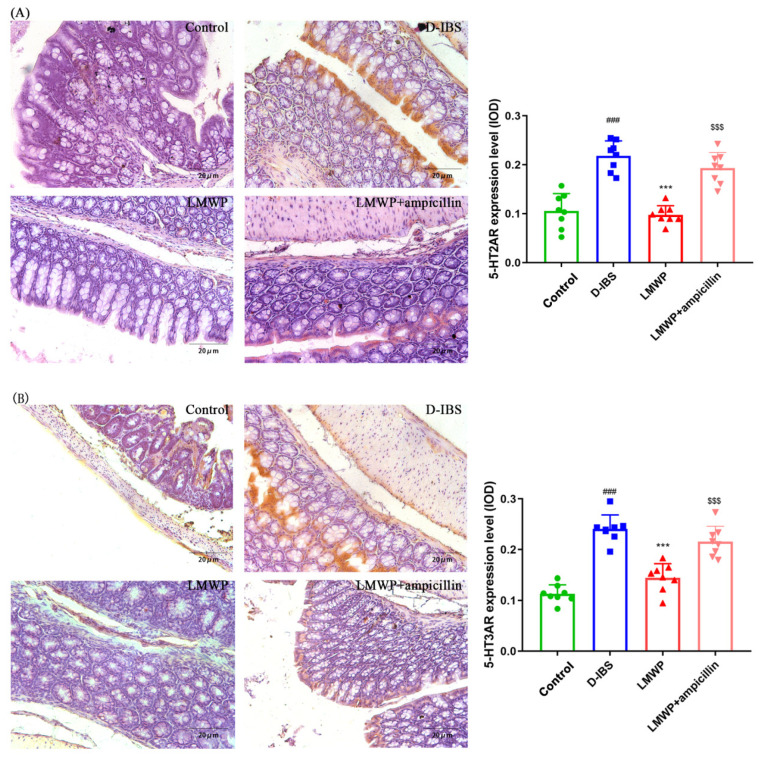
Immunohistochemical staining in the colon. (**A**,**B**) 5-HT2AR and 5-HT3AR. Values are presented as the means ± SD (*n* = 8). ^###^ *p* < 0.001 compared to Control, *** *p* < 0.001 compared to D-IBS and ^$$$^ *p* < 0.001 compared to LMWP (S3-3) from the larvae of *Musca domestica*.

**Figure 6:**
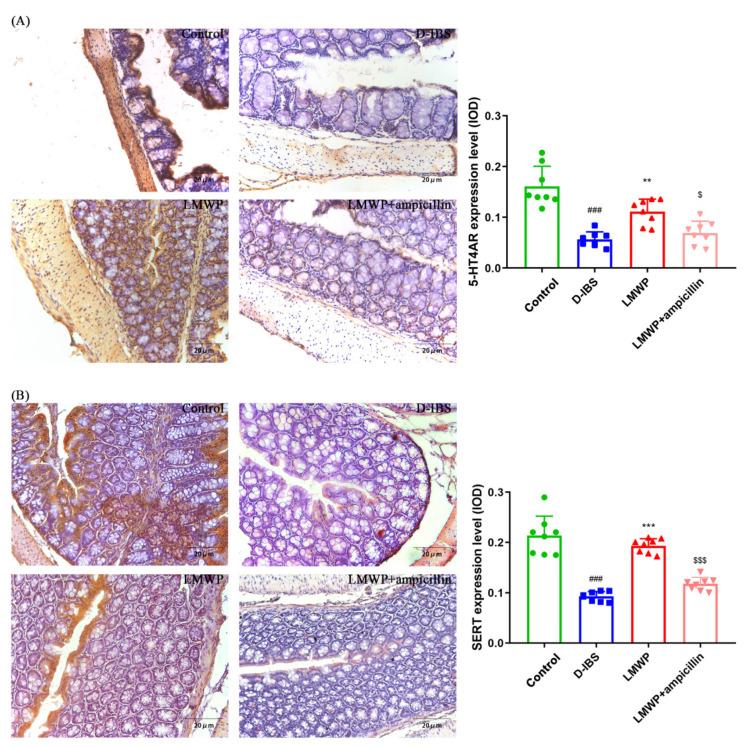
Immunohistochemical staining in the colon. (**A**,**B**) 5-HT4R and SERT. Values are presented as the means ± SD (*n* = 8). ^###^ *p* < 0.001 compared to Control, ** *p* < 0.01 compared to D-IBS, *** *p* < 0.001 compared to D-IBS, ^$^ *p* < 0.05 compared to LMWP (S3-3) from the larvae of *Musca domestica* and ^$$$^ *p* < 0.001 compared to LMWP (S3-3) from the larvae of *Musca domestica*.

The levels of 5-HT in the serum and colon of mice were examined to investigate the effect of LMWP (S3-3) from the larvae of *Musca domestica* by ELISA. The 5-HT concentrations in the serum and colon of mice are shown in Figure 4A,B. Notably, the serum and colon 5-HT concentrations were significantly decreased following treatment with LMWP (S3-3) from the larvae of *Musca domestica*. Additionally, the study used qPCR to examine the expression of the 5-HT2AR, 5-HT3AR, 5-HT4R and SERT genes. The results showed that 5-HT2AR and 5-HT3AR expression in the colon was higher, whereas 5-HT4R and SERT expression was lower in the D-IBS group compared to the Control group (Figure 4C–F). However, LMWP (S3-3) from larvae of *Musca domestica* resulted in a decrease in the expression of both 5-HT2AR and 5-HT3AR (Figure 4C,D). The treatment of larvae of *Musca domestica* with LMWP (S3-3) increased the expression of both 5-HT4R and SERT (Figure 4E,F). However, the LMWP + ampicillin groups increased the 5-HT concentrations in serum and colon tissue, increased 5-HT2AR and 5-HT3AR expression and decreased 5-HT4R and SERT expression (Figure 4). Next, the immunohistological changes in the protein levels of 5-HT2AR, 5-HT3AR, 5-HT4R and SERT were examined in the colon. The results suggested that stress caused an increase in the levels of 5-HT2AR and 5-HT3AR on the membrane surface of the intestinal tissues, which decreased substantially after LMWP (S3-3) treatment (Figure 5A,B). Furthermore, potential immunohistological changes in the protein expressions of SERT in the colon were evaluated. The results showed a significant increase in 5-HT4R and SERT-positive protein expression in the colon in the LMWP (S3-3) group relative to the IBS group (Figure 6A,B). Furthermore, results showed a significant increase in 5-HT2AR, 5-HT3AR protein and a remarkable decrease in 5-HT4R, SERT protein in the colon tissues obtained from mouse in the LMWP (S3-3) + ampicillin group as compared to those in the LMWP (S3-3) group (Figure 5 and Figure 6).

### 3.5. Effects of LMWP (S3-3) from Larvae of Musca domestica on Gut Microbiota in D-IBS Mice

**Figure 7:**
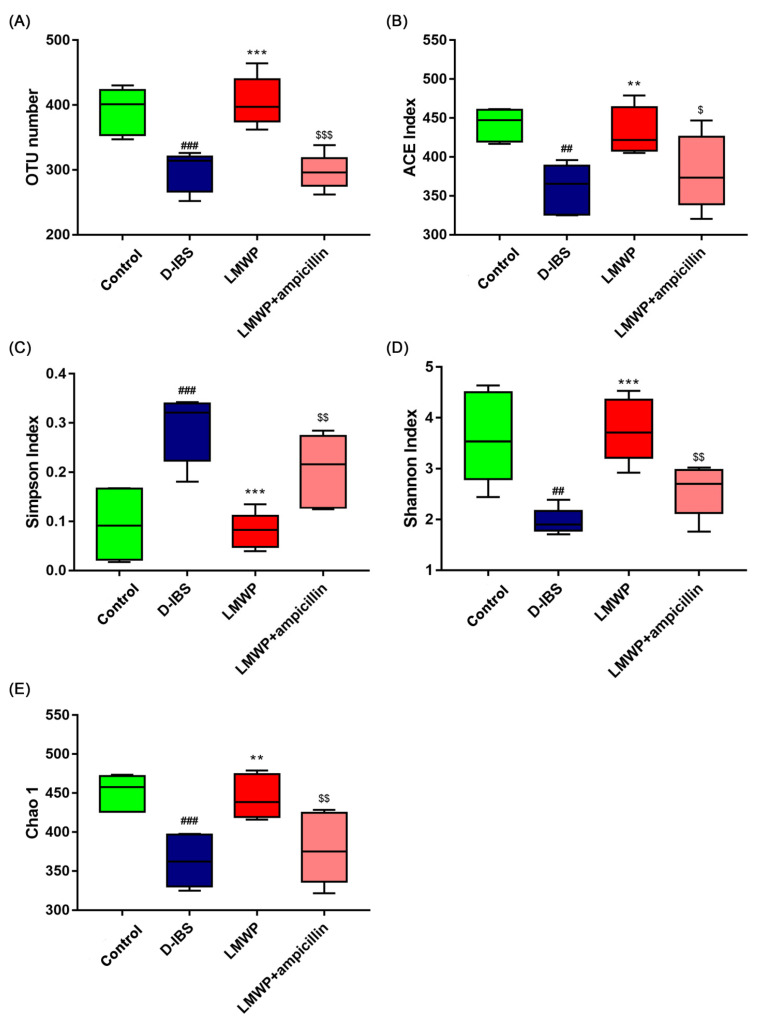
The diversity, richness and structure of the gut microbiota in response to LMWP (S3-3) from the larvae of *Musca domestica* in D-IBS mice. (**A**) The number of OTUs in the gut microbiota. (**B**–**E**) The ACE index, Simpson index, Shannon index and Chao1 index. Values are presented as means ± SD (*n* = 5). ^##^ *p* < 0.01 and ^###^ *p* < 0.001 compared with Control, ** *p* < 0.01 and *** *p* < 0.001 compared with D-IBS, ^$^ *p* < 0.05, ^$$^ *p* < 0.01 and ^$$$^ *p* < 0.001 compared with LMWP (S3-3) from the larvae of *Musca domestica*.

In this study, 16S rDNA sequencing was conducted to determine whether LMWP (S3-3) from larvae of *Musca domestica* influenced the gut microbiota and to define the changes in the composition of gut microbiota. It is noteworthy that the interaction between LMWP (S3-3) from larvae of *Musca domestica* feeding and the gut microbiota has been linked to D-IBS-related metabolic disorder. Therefore, the present study examined the effects of LMWP (S3-3) from the larvae of *Musca domestica* on the composition of gut microbiota by sequencing the V3 + V4 region of bacterial 16S rRNA. The samples were analyzed using high-throughput sequencing, which produced 1,600,995 pairs of raw reads. However, pair-end read alignment and filtering resulted in 1,551,510 clean tags which were subjected to subsequent analysis. All of the effective reads were then clustered into Operational Taxonomic Units (OTUs) based on a 97% similarity level. The dilution curve showed an inflection point at about 1000 and then leveled off, indicating that the sequencing amount of this study was enough to cover almost all bacterial species, indicating that the sample sequence was sufficient. The graded abundance curve indicates that the abundance and evenness of this study are both high, which supports the following data analysis (Supplementary Figures S4 and S5). The functional role of LMWP (S3-3) from the Larvae of *Musca domestica* in improving species richness and diversity of gut microbiota was further supported by increased numbers of OTUs (Figure 7A), ACE (Figure 7B), Shannon (Figure 7D), Chao1 (Figure 7E) index and a lower Simpson index (Figure 7C). However, the LMWP + ampicillin group showed a decrease in species richness and diversity compared to the LMWP group.

**Figure 8:**
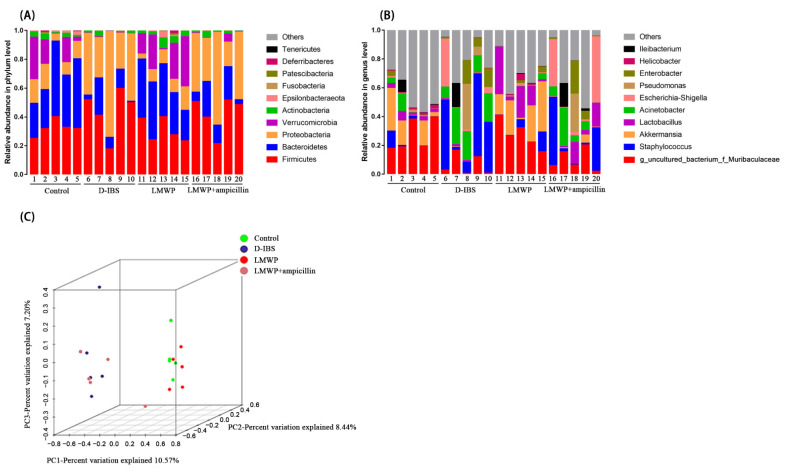
Gut microbiota diversity at the phylum and genus level. (**A**,**B**) Relative abundances of the gut microbiota at the phylum and genus levels. (**C**) Weighted UniFrac-based PCA.

A deeper examination of the microbial community revealed that LMWP (S3-3) from larvae of *Musca domestica* had a significant positive effect on the phylum and genus levels. According to the findings, the phylum level, the 10 most abundant bacteria in the level of phylum could be found and compared in all samples, and the most abundant phyla in all samples were *Firmicutes*, *Bacteroidetes, Proteobacteria* and *Verrucomicrobia* (Figure 8A). At the genus level (Figure 8B), the 10 most abundant bacteria were found and listed in all samples. Additionally, unsupervised multivariate statistical methods such as Principal Component Analysis (PCA) were used to assess structural changes in the gut microbiota. All four groups presented distinct clustering of microbiota composition, and the LMWP group had a similar structure to that of the Control group (Figure 8C). The results of PCA were confirmed through PERMANOVA test, detecting significant differences between groups (Supplementary Figure S7).

**Figure 9:**
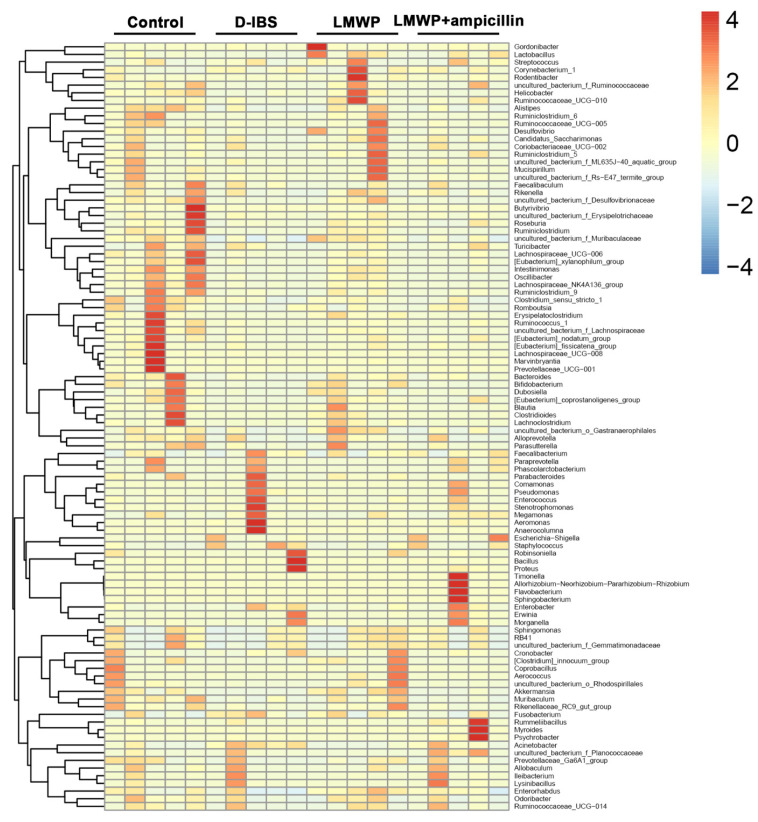
Heatmap of the gut microbiota at the genus level.

Additionally, an overview of the heatmap (Figure 9) suggested a significant effect of LMWP (S3-3) from the larvae of *Musca domestica* on the profile of the gut microbiota. As seen in Figure 9, the abundance increased with the color changing from blue to red; genus-level species clustering analysis was performed according to the distance between each genus-level species. The clustering indicated the similarity of the abundance of different species between samples. The closer the distance between two species was, the shorter the branch length was, indicating that the abundance of these two species was more similar between samples. The clustering revealed the similarity of community composition at each classification level. Therefore, all effective sequences were evaluated using the LEfSe approach to determine the major phenotypes that were significantly altered in response to LMWP (S3-3) of larvae from *Musca domestica* treatment. The findings of the LEfSe analysis revealed the presence of high-dimensional biomarkers in the gut microbiota in each group (Supplementary Figure S8).

**Figure 10:**
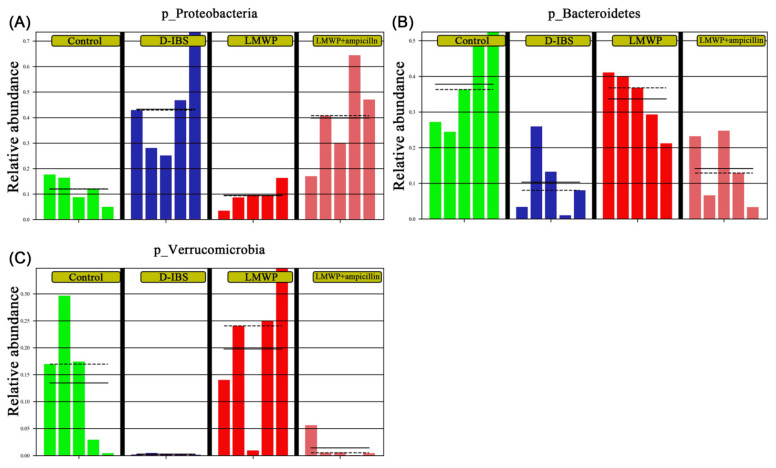
Relative abundances of the gut microbiota at the phylum level. (**A**–**C**) Relative abundances of *Proteobacteria*, *Bacteroidetes* and *Verrucomicrobia*.

**Figure 11:**
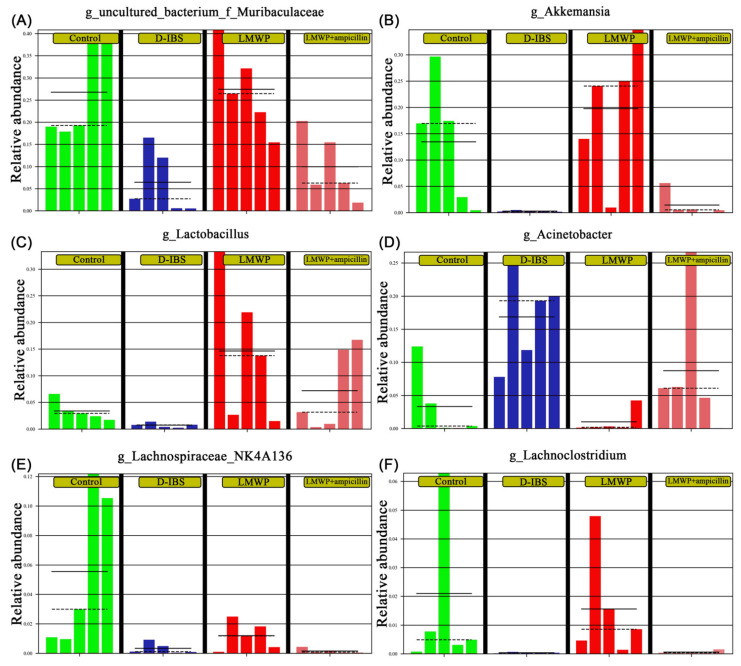
Relative abundances of the gut microbiota at the genus level. (**A**–**F**) Relative abundances of Uncultured_bacterium_f_Muribaculaceae, *Akkermansia*, *Lactobacillus*, *Acinetobacter*, *Lachnospiraceae_NK4A136_group* and *Lachnoclostridium* at the genus level.

Collectively, these findings indicated that treatment with LMWP (S3-3) from larvae of *Musca domestica* reversed D-IBS-induced dysbiosis of the gut microbiota. In comparison to the LMWP group, the LMWP + ampicillin group induced a decrease in *Bacteroidetes* and Verrucomicrobia but increased *Proteobacteria* at the phylum level (Figure 10A–C). The LMWP + ampicillin group induced a decrease in *g_uncultured_bacterium_f_Muribaculaceae*, *Akkermansia*, *Lachnospiraceae_NK4A136_group* and *Lachnoclostridium* but an increase in *Acinetobacter* at the genus level in comparison to the LMWP group (Figure 11A–F).

### 3.6. Potential Correlations among Phenotypes, Molecular Biology Indicators and Gut Microbiota

**Figure 12:**
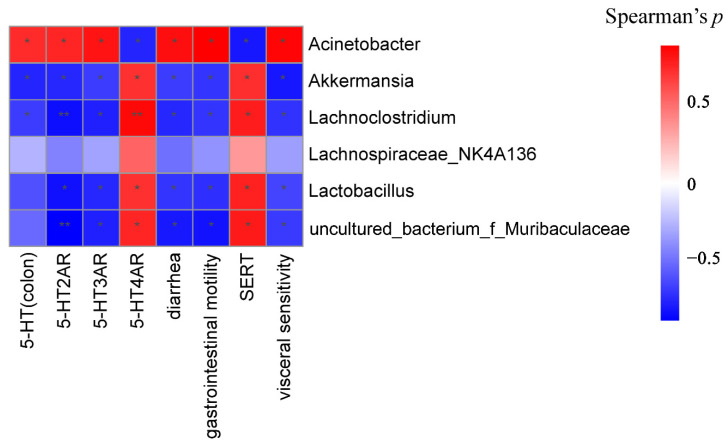
Association map for three-tiered analyses integrating the gut microbiome, D-IBS phenotypes and molecular biology indicator. Scale indicates the level of positive (red) or negative (blue) correlation, * *p* < 0.05 and ** *p* < 0.01.

To further investigate the potential role of the gut microbiota in D-IBS, correlation analyses were performed between diarrhea, gastrointestinal motility, visceral sensitivity, 5-HT, 5-HT2AR, 5-HT3AR, 5-HT4R, SERT and changes in the microbiota (Figure 12). 5-HT, 5-HT2AR and 5-HT3AR were positively correlated with the genus *Acinetobacter*. Negative correlations between 5-HT4R and SERT with *Acinetobacter* were also observed. Additionally, 5-HT, 5-HT2AR and 5-HT3AR were negatively correlated with *Akkermansia* and *Lachnoclostridium*, while 5-HT4R and SERT were positively correlated. *Lactobacillus* was also negatively correlated with 5-HT2AR and 5-HT3AR. *g_uncultured_bacterium_f_Muribaculaceae* and *Lactobacillus* were positively correlated with 5-HT4R and SERT, respectively.

## 4. Discussion

IBS is a chronic relapsing functional gastrointestinal disorder that is characterized by diarrhea and abdominal pain, both of which significantly decrease patients’ quality of life. Currently, available management options mainly focus on symptom alleviation and control of the disease course.

Moreover, our previous study proved intestinal microbiological regulation might be one of the potential antidiarrheal mechanisms of LMWP from the larvae of *Musca domestica* and LMWP (S3-3) successfully isolated and identified from LMWP from the larvae of *Musca domestica* [14]. Therefore, this study focused on determining whether LMWP (S3-3) alleviated D-IBS through regulating gut microbiota. The results suggested that LMWP (S3-3) could alleviate D-IBS by impacting the gut microbiota.

Therefore, the present study established a restraint stress model of D-IBS in mice. After 4 weeks, mice in the D-IBS group had a lower body weight and a higher diarrheal index, indicating that they were experiencing diarrhea. Interestingly, LMWP (S3-3) from the larvae of *Musca domestica* decreased diarrhea caused by D-IBS, indicating that LMWP (S3-3) from the larvae of *Musca domestica* was capable of relieving diarrhea in these mice. Additionally, LMWP (S3-3) from the larvae of *Musca domestica* reduced the AWR scores in D-IBS mice, implying that it might be able to alleviate intestinal visceral hypersensitivity in the animals. LMWP (S3-3) from the larvae of *Musca domestica* also inhibited colonic motility and prolonged gastrointestinal transit in mice. These results demonstrated that LMWP (S3-3) from the larvae of *Musca domestica* were beneficial for gastrointestinal motility and possessed antinociceptive properties. Nonetheless, cotreatment with antibiotics (ampicillin + LMWP (S3-3) from the larvae of *Musca domestica*) significantly reduced the beneficial effects of LMWP (S3-3) from larvae of *Musca domestica* against D-IBS. Metabolic products from gastrointestinal microbiota fermentation, such as SCFAs, or peptides can act on the ENS and affect gut transit [34]. The neuroendocrine system of the gut has also been shown to interact with microbiota [35] via 5-HT [36]. 5-HT is produced in both the ENS and CNS and is a key neurotransmitter that plays a pivotal role in mediating motor and secretory responses in the ENS [37]. 5-HT stimulates local enteric nervous reflexes to initiate secretion and propulsive motility and acts on vagal afferents to modulate contractile activities [37].

IBS is a complex disorder characterized by changes in sensation, secretion and gastrointestinal motility. 5-HT is a critical signaling molecule in the gut that targets enterocytes, smooth muscles and enteric neurons, activating both intrinsic and extrinsic primary afferent neurons to initiate peristaltic and secretory reflexes, as well as transmitting information to the central nervous system. Therefore, the serum and colon 5-HT concentrations were determined in this study by ELISA. Additionally, the data revealed that LMWP (S3-3) from the larvae of *Musca domestica* were capable of lowering 5-HT levels in the serum and colon. 5-HT has been shown to play an important role in regulating intestinal motility [38]. However, excessive 5-HT production induces high visceral sensitivity, and this is an important mechanism of D-IBS [38,39]. 5-HT has a remarkable range of effects that are attributable to the existence of multiple receptor subtypes on enteric neurons, enterochromaffin cells (EC cells), GI smooth muscle and probably enterocytes and immune tissue. 5-HT receptors are now classified into seven families and subtypes, with 5-HT2AR, 5-HT3AR and 5-HT4R known to affect gut motor functions. Additionally, 5-HT is inactivated by the SERT-mediated uptake into enterocytes or neurons. Therefore, the genes and proteins of the 5-HT-related pathway were detected by qPCR and IHC in this study. The present study demonstrated that LMWP (S3-3) from the larvae of *Musca domestica* could decrease the levels of 5-HT in the colon of D-IBS model mice by down-regulating the expression of 5-HT2AR, 5-HT3AR and up-regulating the expression of 5-HT4R and SERT. Furthermore, the binding of 5-HT and 5-HT2AR was reported to block voltage-gated K+ channels and increase visceral sensitivity by generating enteric neuron excitation [40]. Moreover, 5-HT3R are found in a variety of locations, including peripheral primary sensory nerve endings, autonomic preganglionic and postganglionic neurons, the central nervous system and the lower brainstem, among other areas. Notably, activation of the 5-HT3R, which has excitatory effects, mediates the rapid activation of sensory afferents, hence enhancing nerve-mediated gastrointestinal motility and secretion. Additionally, it generates visceral pain stimuli, which results in abdominal pain [41]. Furthermore, 5-HT4R is positively associated with adenylate cyclase, which is located on the mesenteric plexus neurons [42]. 5-HT4R has also been identified in the intestinal primary afferent neurons [43] and was shown to be involved in the peristaltic reflex [38,39]. On the other hand, Serotonin Reuptake Transporter (SERT) is a highly regulated protein, located on the membrane of intestinal epithelial cells and is involved in the reuptake of 5-HT [44]. Moreover, excess 5-HT is often transported into epithelial cells via SERT and inactivated there. Therefore, inhibiting the expression of SERT can induce the sensitivity of primary neurons to 5-HT, and this can, in turn, enhance visceral sensitivity [44,45].

The findings indicated that the levels of 5-HT in the serum and colon were elevated in D-IBS mice and that LMWP (S3-3) from the larvae of *Musca domestica* could decrease the levels of 5-HT in the serum and colon of D-IBS mice. 5-HT, which is primarily produced in the gut, regulates intrinsic reflexes (e.g., stimulates motility, secretion and vasodilation) and may contribute to the development of diarrhea by promoting inflammation [46,47]. The EC cells are mucosal sensory cells that release mediators (5-HT, among others) in response to chemical or mechanical stimulation [48]. It is hypothesized that excessive release of 5-HT from EC cells may contribute to diarrhea in IBS patients [48]. Rapid intake of 5-HT occurs via a selective SERT transporter that regulates 5-HT in the gut [48]. The enteric nervous system (ENS) is also involved in intestinal absorption and secretion, and diarrhea has been associated with decreased absorption of ions and/or solutes and water [49]. Numerous studies indicate that 5-HT and the ENS may play an important role in the pathophysiology of IBS and perhaps in diarrhea [48]. The findings also demonstrated that the SERT levels in the colon of D-IBS mice were decreased and LMWP (S3-3) from the larvae of *Musca domestica* could enhance the levels of SERT in the colon of D-IBS mice SERT decrease can affect motility and thus contribute to diarrhea [48,50]. Therefore, LMWP (S3-3) from the larvae of *Musca domestica* may be used to treat diarrhea in D-IBS mice by regulating 5-HT and SERT levels.

In our view, LMWP (S3-3) supplemented intestinal nutrition and produced prebiotics, then regulated gut microbiota through prebiotics, and then regulated SCFAs, which affect release of 5-HT, through gut microbiota [51,52,53]. In this study, changes in the composition of the microbiota were examined using high-throughput sequencing. The results demonstrated that the alpha diversity of the gut microbiota was reduced in the D-IBS group, which had a lower Shannon index, ACE index and Chao1 index and a higher Simpson index than the Control group. Nevertheless, treatment with LMWP (S3-3) from the larvae of *Musca domestica* was able to restore diversity. Additionally, PCoA indicated significant distances between each group, indicating that the beta diversity of gut microbiota was different in D-IBS model mice and LMWP (S3-3)-treated mice. According to the findings, the relative abundance of *Lactobacillus* was decreased in D-IBS model mice compared to Control mice. Moreover, treatment with LMWP (S3-3) from the larvae of *Musca domestica* increased the relative abundance of *Lactobacillus*, which has previously been shown to have positive therapeutic benefits on IBS [54,55]. Short-chain Fatty Acids (SCFAs) are the primary metabolites of the gut microbiota and can boost the growth of *Lactobacillus*. They are also critical signaling molecules that affect intestinal function, and abnormal changes in SCFA levels have been associated with IBS. Additionally, it was demonstrated that intestinal microbiota imbalances in IBS patients have a direct effect on the normal signaling interactions between intestinal microbiota, SCFAs and intestinal epithelial cells, resulting in a low inflammatory response, increased permeability of the intestinal epithelial barrier and hypermotility [56]. We also found that LMWP (S3-3) increased related SCFA concentration, such as propionate and butyrate (Supplementary Figure S6, Supplementary Methods). On the contrary, the current study found a significant increase in the abundance of *Akkermansia* after treatment with LMWP (S3-3) from the larvae of *Musca domestica*. *Akkermansia* is a probiotic belonging to the *Verrucomicrobia* phylum and is involved in nutrition metabolism. Recent studies also indicate that *Akkermansia* improves metabolic health and protects against obesity, diabetes and inflammation in the intestinal tract of rodents by interacting with intestinal epithelial cells [56,57]. Additionally, *Akkermansia muciniphila* is the type species of the genus *Akkermansia*, which was first proposed in 2004 as a mucin-degrading, anaerobic Gram-negative bacterium that resides in the mucus layer [58]. Notably, the mucus layer lining the intestinal tract serves as a lubricant and physiological barrier between the luminal contents and mucosal surface. Furthermore, the presence of *A. muciniphila* in IBS mice may play an important role in preserving the integrity of the mucin layer. However, it is unclear whether LMWP (S3-3) from the larvae of *Musca domestica* increases the abundance of *A. muciniphila* by providing the primary source of energy for this bacterium, thereby favoring its growth. Additionally, it is unknown if an increase in *A. muciniphila* increases mucus production and degradation. However, it was discovered that treatment with LMWP (S3-3) from the larvae of *Musca domestica* inhibited the proliferation of *Acinetobacter*, which are mostly opportunistic microbes whose population increased significantly in mice with diarrhea [59]. According to the findings, the relative abundance of *g_uncultured_bacterium_f_Muribaculaceae* was decreased in D-IBS model mice compared to Control mice, which belong to *Muribaculaceae*. LMWP (S3-3) also increased the relative abundance of *g_uncultured_bacterium_f_Muribaculaceae*. Schmidt et al. also found that the abundance of *Muribaculaceae* was strongly correlated with the concentration of propionate belonging to SCFAs [60].

The findings also showed that gut microbiota play a key role in D-IBS by enhancing the function of LMWP (S3-3) from the larvae of *Musca domestica*. This is because when LMWP (S3-3) from larvae of *Musca domestica* were combined with an antibiotic, the regulatory effect on physiological conditions, diarrhea, gastrointestinal motility, visceral sensitivity, colon histology, levels of 5-HT, expression of associated pathway genes and proteins, and gut microbiota were significantly reduced. Ampicillin is a β-lactam antibiotic that has the potential to disrupt gut microbiota and cause diarrhea [61,62]. The mechanism of ampicillin-induced diarrhea may be related to disruption to the normal composition and functional attributes of the gut microbiota [63], where 5-HT was related to diarrhea [64]. Compared with the LMWP group, for LMWP (S3-3) from larvae of *Musca domestica* that were combined with ampicillin, the regulatory effect on physiological conditions, diarrhea, gastrointestinal motility, visceral sensitivity, colon histology, levels of 5-HT, expression of associated pathway genes and proteins, and gut microbiota were significantly reduced, which suggested that the changes in gut microbiota composition might alter colonic motility. Gut microbiota regulated SCFAs, which affect release of 5-HT [51,52]. 5-HT has been shown to play an important role in regulating GI motility [38]. Additionally, several studies have revealed that the germ-free condition is characterized by increased plasma 5-HT concentrations. Plasma 5-HT levels are thought to be mostly derived from intestinal EC cells of the gut [65,66].

In this study, these data suggested that LMWP (S3-3) from the larvae of *Musca domestica* had an obvious protective effect on D-IBS through regulating 5-HT-pathway-related genes and proteins and adjusting gut microbiota.

## 5. Conclusions

**Figure 13:**
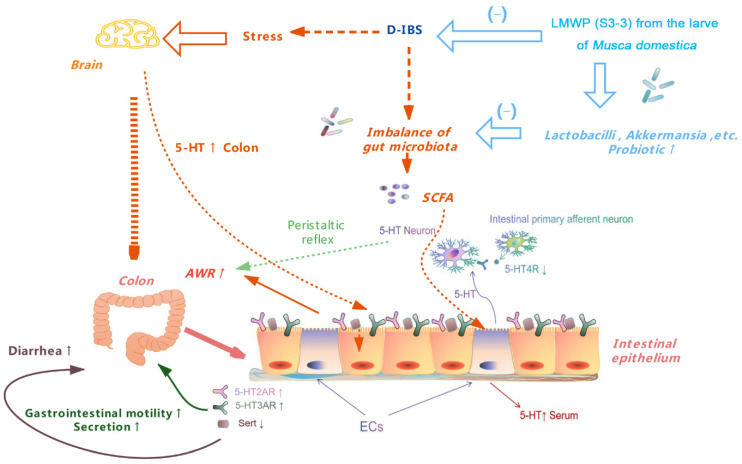
A schematic presentation of the therapeutic effect of LMWP (S3-3) from the larvae of *Musca domestica* on D-IBS.

According to the current study’s findings, treatment with LMWP (S3-3) from the larvae of *Musca domestica* regulates gut microbiota by increasing the relative abundance of *Akkermansia* and *Lactobacillus*. Additionally, LMWP (S3-3) treatment decreases *Acinetobacter* levels, resulting in favorable benefits against diarrhea, increased visceral sensitivity and excessive gastrointestinal motility. This also regulates the 5-HT levels in serum and colon as well as the expression of 5-HT-pathway-related genes and proteins (Figure 13). The data of this study suggested that LMWP (S3-3) from larvae of *Musca domestica* had an obvious protective effect on D-IBS, potentially by adjusting gut microbiota, down-regulating 5-HT, 5-HT2AR and 5-HT3AR, up-regulating 5-HT4R and SERT, relieving diarrhea, decelerating the gastrointestinal motility and alleviating intestinal visceral hypersensitivity. These findings could enhance our understanding of the effect and mechanism of LMWP (S3-3) from larvae of *Musca domestica* on D-IBS and contribute to developing effective therapies in the future.

## Figures and Tables

**Table 1:** The primers used in this experiment.

Gene Name	Primer Sequence (from 5’ End to 3’ End)	Product Size (bp)
5-HT2AR-F	ACCGCTTTGGCAGTTTT	140
5-HT2AR-R	GCGTTGAGGTGGCTTATT	
5-HT3AR-F	GCAACCCCAGTCTCTTTGT	143
5-HT3AR-R	GCTTGACGCCCTGATAAGT	
5-HT4R-F	CATGCCCAGCAGATACAG	147
5-HT4R-R	GAAACAGAAGCAGCCCAT	
SERT-F	CTCCTCCCCTCTAAGCCA	185
SERT-R	CCTCCTTCCTCTCCTCACA	
GAPDH-F	GATGGACACATTGGGGTT	148
GAPDH-R	AAAGCTGTGGCGTGATG	

## Data Availability

The original contributions presented in the study are publicly available. These data can be found here: https://www.ncbi.nlm.nih.gov/bioproject/900466 (accessed on 23 February 2022), BioProject ID is PRJNA900466.

## Supplementary Materials

The following supporting information can be downloaded at: https://www.mdpi.com/article/10.3390/molecules27144517/s1, Figure S1: The retention time in the RP-HPLC; Figure S2: The molecular weight of LMWP(S3-3) was detected by MALDI-TOF spectromete; Figure S3: *N*-terminal sequence of LMWP(S3-3) were determined by Edman degradation; Figure S4: OTU Rarefaction Curve; Figure S5: Rank Abundance Curve; Figure S6: SCFA variations; Figure S7: Permanova test; Figure S8: LEfSe analysis results; Table S1: LMWP(S3-3) HPLC peak area integral results. Table S2: Association map data.

## Sample Availability

Samples of the compounds LMWP(S3-3) are available from the authors.
