# Supplementary material for: LMWP (S3-3) from the Larvae of Musca domestica Alleviate D-IBS by Adjusting the Gut Microbiota
Source: Molecules. 2022 Jul 15;27(14):4517. doi: 10.3390/molecules27144517 (PMC9324334; doi:10.3390/molecules27144517)
Supplement: Supplementary file 1 [file molecules-27-04517-s001.zip › molecules-1741405-SI.pdf]

# *Supplementary Material*

## 1 Supplementary Figures and Tables

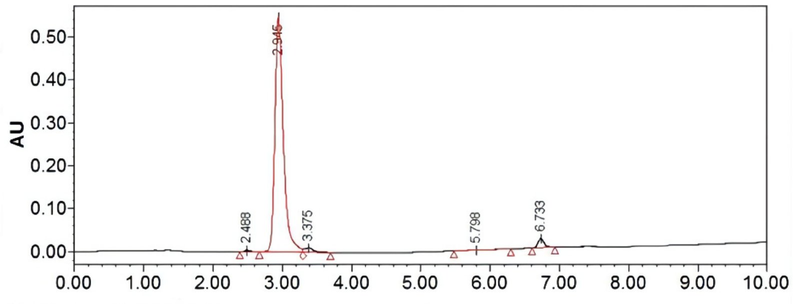


**Supplementary Figure S1 | The retention time in the RP-HPLC.** The retention time of LMWP(S3-3) peak in the RP-HPLC is 2.9454 min.

**Supplementary Table S1 | LMWP(S3-3) HPLC peak area integral results.** The purity of LMWP(S3-3) was 94.70% which calculated according to the area of the peaks.

| | Retention time (min) | Peak area (AU*S) | % Peak area | Peak height (μU) | Integral type |
|---|---|---|---|---|---|
| 1 | 2.488 | 21453 | 0.44 | 3668 | BB |
| 2 | 2.945 | 4539208 | 94.70 | 545313 | BV |
| 3 | 3.375 | 88705 | 1.81 | 9926 | VB |
| 4 | 5.798 | 24107 | 0.49 | 896 | BB |
| 5 | 6.733 | 125270 | 2.56 | 21281 | BB |

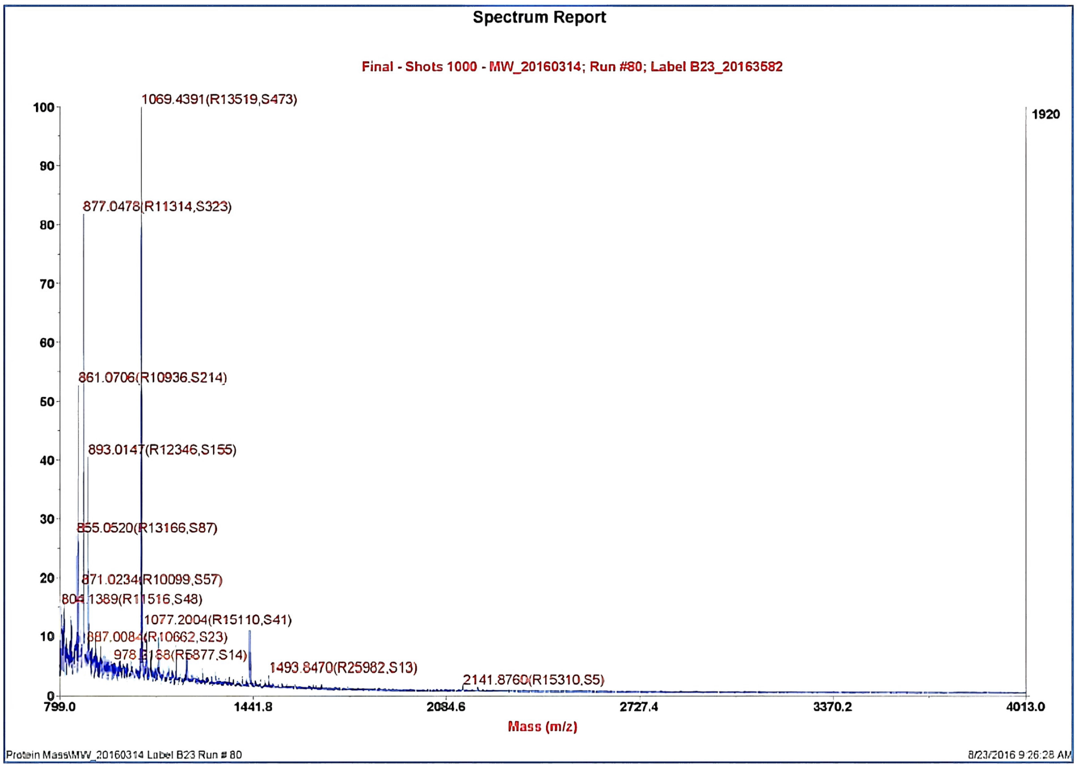


**Supplementary Figure S2 | The molecular weight of LMWP(S3-3) was detected by MALDI-TOF spectromete.** The data were analyzed by Series Explorer V3.5 software, results showed exact molecular weight of S3-3 was 1069.4391D

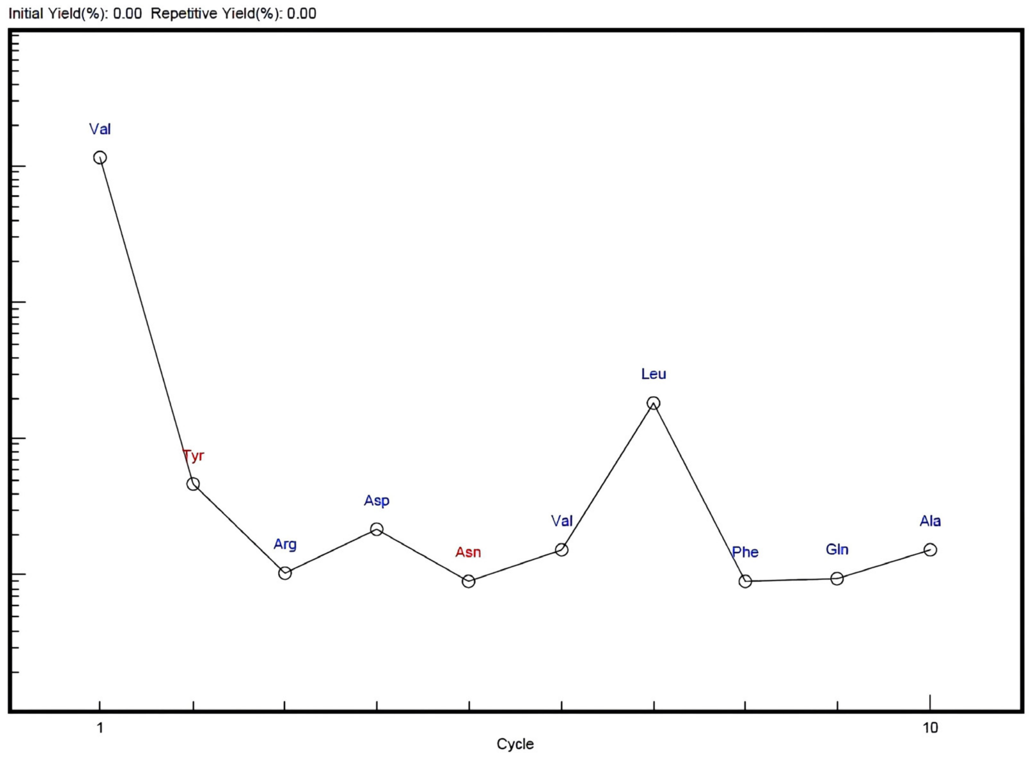


**Supplementary Figure S3 | N-terminal sequence of LMWP(S3-3) were determined by Edman degradation.** The 10 amino acid sequences of S3-3 were VYRDNVLFQA (Val-Tyr-Arg-Asp-Asn-Val-Leu-Phe-Gln-Ala).

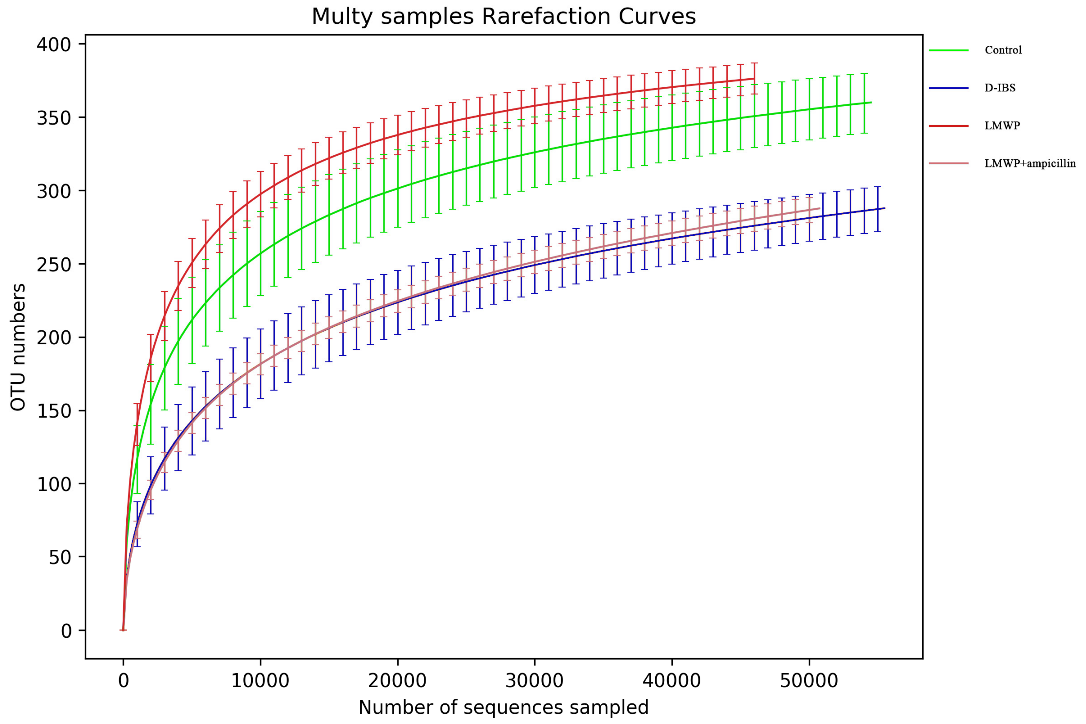


**Supplementary Figure S4 | OTU Rarefaction Curve.** The dilution curve showed an inflection point at about 1000 and then leveled off, indicating that the sequencing amount of this study was enough to cover almost all bacterial species, indicating that the sample sequence was sufficient.

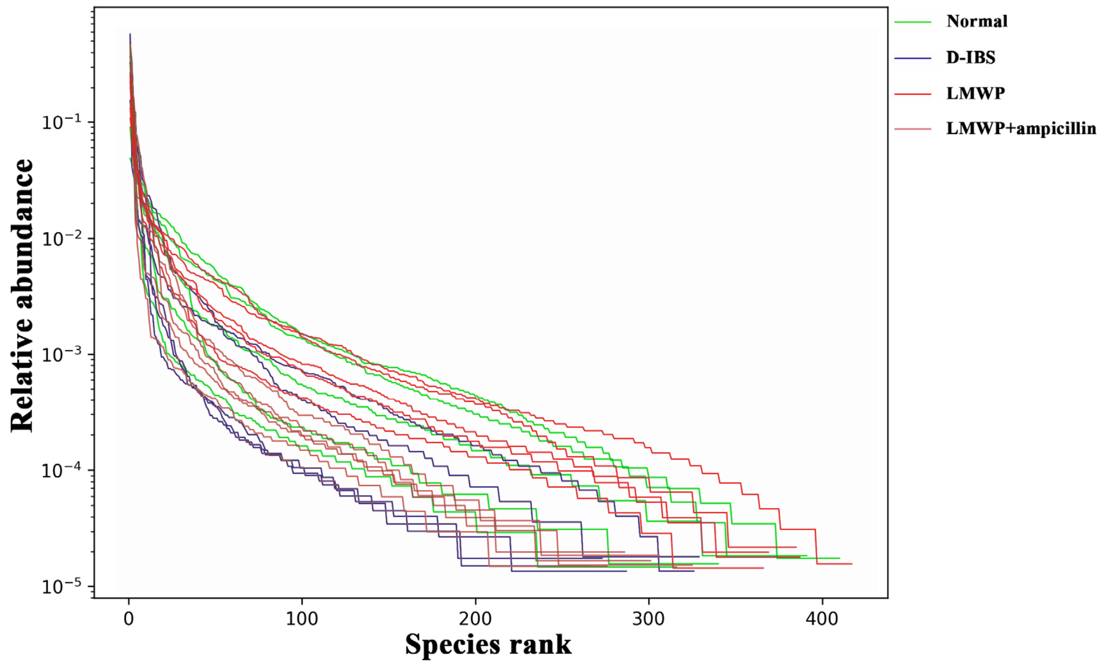


**Supplementary Figure S5 | Rank Abundance Curve.** the graded abundance curve indicates that the abundance and evenness of this study are both high.

**Supplementary Table S2 | Association map data.** Association map data for analyses integrating the gut microbiome, diarrhea, gastrointestinal motility, visceral sensitivity, 5-HT, 5-HT2AR, 5-HT3AR, 5-HT4R and SERT. $|r| \geqslant 0.5$, $*p < 0.05$, $**p < 0.01$

| category1 | category2 | Correlation | *p*-value | Significance |
|---|---|---|---|---|
| 5-HT(colon) | uncultured_bacterium_f_S24-7 | -0.527272727 | 0.122803188 | |
| 5-HT(colon) | Akkermansia | -0.76969697 | 0.013671782 | * |
| 5-HT(colon) | Acinetobacter | 0.721212121 | 0.024194588 | * |
| 5-HT(colon) | Lactobacillus | -0.612121212 | 0.066469137 | |
| 5-HT(colon) | Lachnoclostridium | -0.684848485 | 0.035091538 | * |
| 5-HT(colon) | Lachnospiraceae_NK4A136 | -0.272727273 | 0.448272159 | |
| 5-HT3AR | uncultured_bacterium_f_S24-7 | -0.781818182 | 0.011651268 | * |
| 5-HT3AR | Akkermansia | -0.684848485 | 0.035091538 | * |
| 5-HT3AR | Acinetobacter | 0.793939394 | 0.009844136 | ** |
| 5-HT3AR | Lactobacillus | -0.757575758 | 0.015920829 | * |
| 5-HT3AR | Lachnoclostridium | -0.781818182 | 0.011651268 | * |
| 5-HT3AR | Lachnospiraceae_NK4A136 | -0.333333333 | 0.348846244 | |
| visceral sensitivity | uncultured_bacterium_f_S24-7 | -0.687835939 | 0.027915724 | * |
| visceral sensitivity | Akkermansia | -0.812897019 | 0.004249197 | ** |
| visceral sensitivity | Acinetobacter | 0.844162289 | 0.002128395 | ** |
| visceral sensitivity | Lactobacillus | -0.656570669 | 0.03919122 | * |
| visceral sensitivity | Lachnoclostridium | -0.719101209 | 0.01908823 | * |
| visceral sensitivity | Lachnospiraceae_NK4A136 | -0.34391797 | 0.330526431 | |
| 5-HT2AR | uncultured_bacterium_f_S24-7 | -0.893621149 | 0.000491873 | *** |
| 5-HT2AR | Akkermansia | -0.75988193 | 0.010758005 | * |
| 5-HT2AR | Acinetobacter | 0.735565708 | 0.015323456 | * |

| | | | | |
|---|---|---|---|---|
| 5-HT2AR | Lactobacillus | -0.832830595 | 0.002777889 | ** |
| 5-HT2AR | Lachnoclostridium | -0.83890965 | 0.002413988 | ** |
| 5-HT2AR | Lachnospiraceae_NK4A136 | -0.443771047 | 0.198893442 | |
| gastrointestinal motility | uncultured_bacterium_f_S24-7 | -0.83030303 | 0.005556805 | ** |
| gastrointestinal motility | Akkermansia | -0.709090909 | 0.027514119 | * |
| gastrointestinal motility | Acinetobacter | 0.866666667 | 0.002681415 | ** |
| gastrointestinal motility | Lactobacillus | -0.733333333 | 0.021166481 | * |
| gastrointestinal motility | Lachnoclostridium | -0.709090909 | 0.027514119 | * |
| gastrointestinal motility | Lachnospiraceae_NK4A136 | -0.381818182 | 0.278965216 | |
| diarrhea(loose stool index) | uncultured_bacterium_f_S24-7 | -0.806060606 | 0.008235571 | ** |
| diarrhea(loose stool index) | Akkermansia | -0.684848485 | 0.035091538 | * |
| diarrhea(loose stool index) | Acinetobacter | 0.818181818 | 0.006811133 | ** |
| diarrhea(loose stool index) | Lactobacillus | -0.709090909 | 0.027514119 | * |
| diarrhea(loose stool index) | Lachnoclostridium | -0.757575758 | 0.015920829 | * |
| diarrhea(loose stool index) | Lachnospiraceae_NK4A136 | -0.503030303 | 0.143366802 | |
| 5-HT4AR | uncultured_bacterium_f_S24-7 | 0.729486652 | 0.01664688 | * |
| 5-HT4AR | Akkermansia | 0.699091375 | 0.024470991 | * |
| 5-HT4AR | Acinetobacter | -0.765960985 | 0.00978704 | ** |
| 5-HT4AR | Lactobacillus | 0.699091375 | 0.024470991 | * |
| 5-HT4AR | Lachnoclostridium | 0.83890965 | 0.002413988 | ** |
| 5-HT4AR | Lachnospiraceae_NK4A136 | 0.516719712 | 0.126197367 | |
| SERT | uncultured_bacterium_f_S24-7 | 0.77204004 | 0.008879537 | ** |
| SERT | Akkermansia | 0.705170431 | 0.022737737 | * |

| | | | | |
|---|---|---|---|---|
| SERT | Acinetobacter | -0.814593429 | 0.004106132 | ** |
| SERT | Lactobacillus | 0.753802874 | 0.011794786 | * |
| SERT | Lachnoclostridium | 0.765960985 | 0.00978704 | ** |
| SERT | Lachnospiraceae_NK4A136 | 0.340427104 | 0.335782263 | |

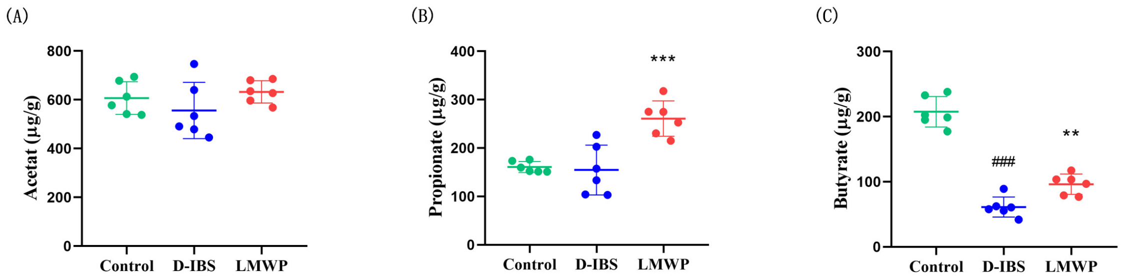


**Supplementary Figure S6 | SCFA variations.** LMWP(S3-3) enriched the abundance of propionate concentration and butyrate concentration in mice.

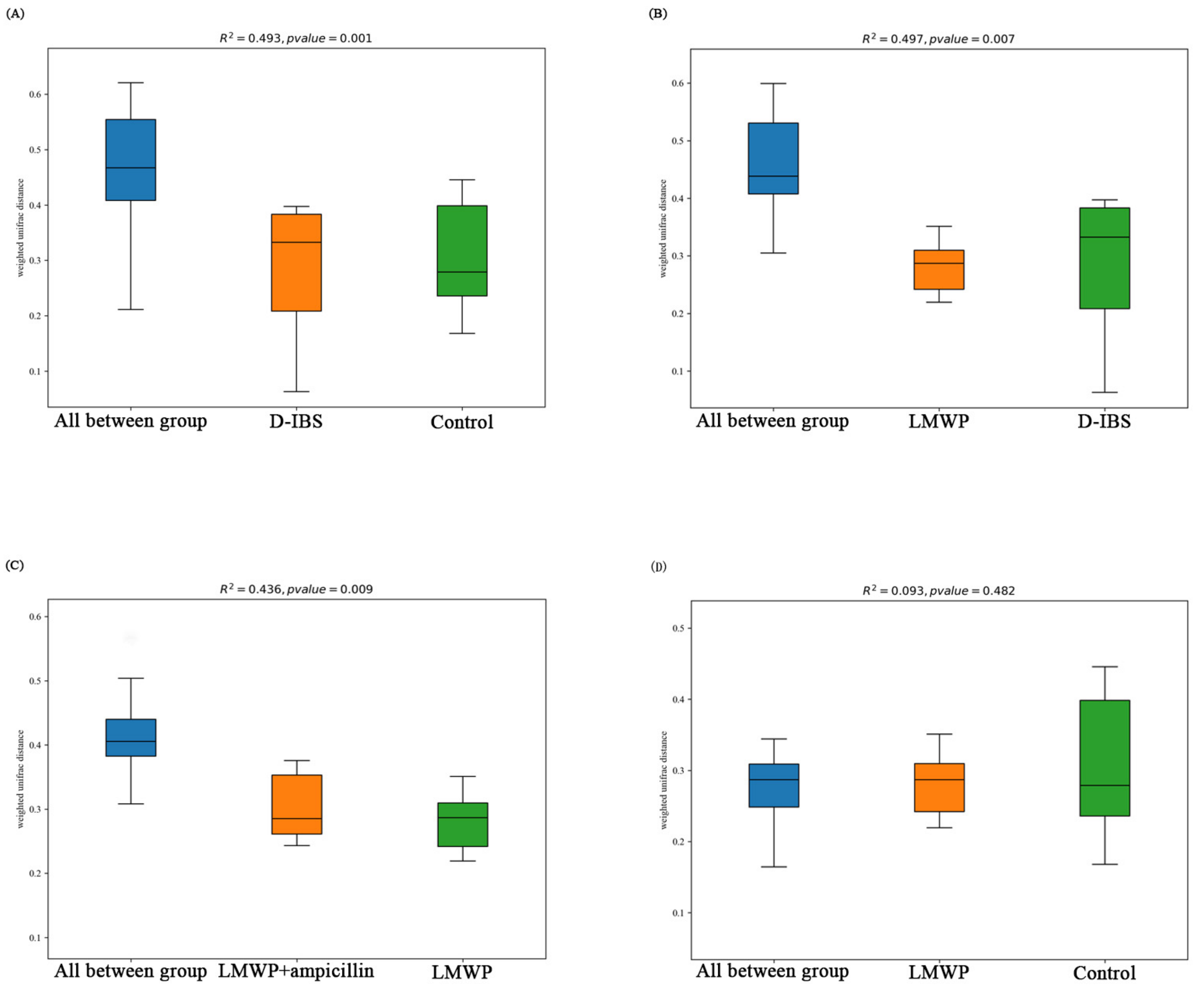


**Supplementary Figure S7 | Permanova test.** D-IBS group vs Control group, LMWP group vs D-IBS group, LMWP+ampicillin group vs LMWP group and LMWP group vs Control group were tested in PERMANOVA.

**Supplementary Methods | Measurements of colon content SCFAs.**

The quantification analysis of colon content SCFAs was determined by gas chromatography-mass spectrometry (GC–MS) using Thermo TRACE-ISQ7000. Briefly, 100 mg sample was weighed and mixed with 1 mL Ethanol solution（containing 0.5% HCl）. Next, the extraction was sonicated for 30 min and centrifuged for 10 min (4 °C at 14,000 rpm). The supernatant was taken over the organic filter membrane and analyzed by GC-MS. TG-WAXMS capillary column (30 m*0.25 mm*0.25 μm) was used for chromatographic separation. The temperature of inle were 250℃, no diversion.. Program heating initial temperature was 50℃ and incubated for 2 min, then raise the temperature to 120℃ at 15℃/min, and then raise the temperature to 170℃ at 5℃/min, then up to 240°C at 15°C / min for 3 min. The helium carrier flow rate was 1.0 mL/ min. Electron bombardment ionization (EI) source, full sweep and SIM scanning mode, electron energy of 70eV. Data handing was performed with an Dionex Chromeleon 7.2 chromatographic data system (Thermo Scientific, USA).

(A)

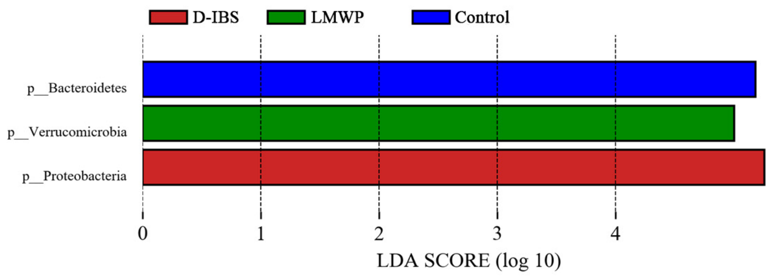


(B)

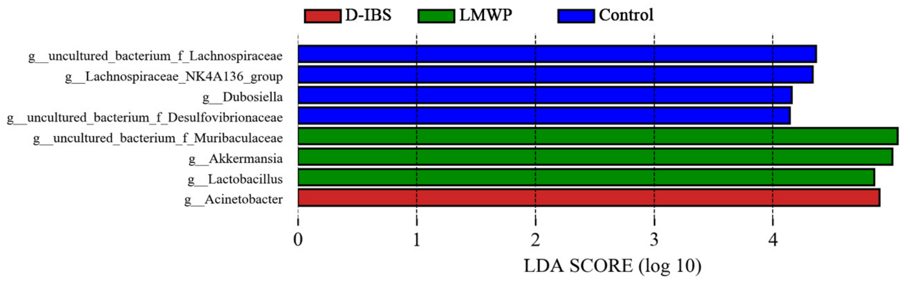


**Supplementary Figure S8 | LEfSe analysis results.** In phylum level, *Bacteroidetes, Verrucomicrobia* and *Proteobacteria* displayed significant differences among 4 groups. In genus level, *g_uncultured_bacterium_f_Muribaculaceae*, *g_uncultured_bacterium_f_Lachnospiraccae, Lachnospiraceae_NK4A136_group*, *Lactibacillus*, *Akkermansia*, *Acinetobacter* and *Lachnoclostridium* displayed significant differences among 4 groups.
